# KCNQ channel openers reverse depressive symptoms via an active resilience mechanism

**DOI:** 10.1038/ncomms11671

**Published:** 2016-05-24

**Authors:** Allyson K. Friedman, Barbara Juarez, Stacy M. Ku, Hongxing Zhang, Rhodora C. Calizo, Jessica J. Walsh, Dipesh Chaudhury, Song Zhang, Angel Hawkins, David M. Dietz, James W. Murrough, Maria Ribadeneira, Erik H. Wong, Rachael L. Neve, Ming-Hu Han

**Affiliations:** 1 Department of Pharmacology and Systems Therapeutics, Icahn School of Medicine at Mount Sinai, New York, New York 10029, USA; 2 Department of Biological Sciences, Hunter College, Biology and Biochemistry PhD Program, Graduate Center, The City University of New York, New York, New York 10065, USA; 3 Graduate School of Biomedical Sciences, Icahn School of Medicine at Mount Sinai, New York, New York 10029, USA; 4 Fishberg Department of Neuroscience, Icahn School of Medicine at Mount Sinai, New York, New York 10029, USA; 5 Department of Psychiatry, Icahn School of Medicine at Mount Sinai, New York, New York 10029, USA; 6 Friedman Brain Institute, Icahn School of Medicine at Mount Sinai, New York, New York 10029, USA; 7 CNS Pain Innovative Medicine Unit, AstraZeneca Pharmaceuticals, Wilmington, Delaware 19850, USA; 8 McGovern Institute for Brain Research, Department of Brain and Cognitive Sciences, Massachusetts Institute of Technology, Cambridge, Massachusetts 02139, USA

## Abstract

Less than half of patients suffering from major depressive disorder, a leading cause of disability worldwide, achieve remission with current antidepressants, making it imperative to develop more effective treatment. A new therapeutic direction is emerging from the increased understanding of natural resilience as an active stress-coping process. It is known that potassium (K^+^) channels in the ventral tegmental area (VTA) are an active mediator of resilience. However, no druggable targets have been identified to potentiate active resilience mechanisms. In the chronic social defeat stress model of depression, we report that KCNQ-type K^+^ channel openers, including FDA-approved drug retigabine (ezogabine), show antidepressant efficacy. We demonstrate that overexpression of KCNQ channels in the VTA dopaminergic neurons and either local infusion or systemic administration of retigabine normalized neuronal hyperactivity and depressive behaviours. These findings identify KCNQ as a target for conceptually novel antidepressants that function through the potentiation of active resilience mechanisms.

## Abstract (web summary)

Potassium channels in the ventral tegmental area are known to regulate resilience against stress-induced depression. Here, the authors show over expression of KCNQ3 channels in VTA dopaminergic neurons or treatment with KCNQ channel openers normalizes depressive behaviours in mouse models.

## Graphical abstract

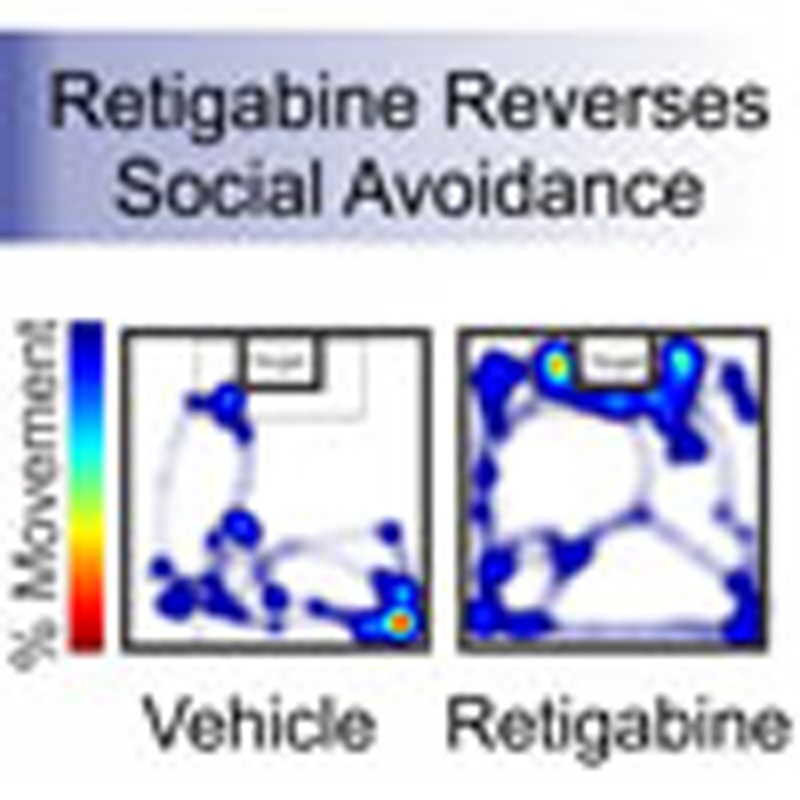

We previously demonstrated that chronic social defeat stress provides an excellent model of both stress-induced depression and resilience^1^,^2^,^3^,^4^,^5^,^6^. Interestingly, resilient mice regulate more genes in the ventral tegmental area (VTA) than the susceptible (depressed) subpopulation. This supports the new concept that resilience is not a passive absence of stress-induced depression, but actually is an active stress-coping process by which resilient mice homeostatically maintain their healthy behaviours^1^,^3^. Consistently, at the functional level, resilient mice use more ion channels, including actively regulating several K^+^ channels, to stabilize VTA dopaminergic (DA) neurons and counteract the pathologic hyperactivity of these neurons seen in the susceptible subgroup^3^,^7^,^8^, further supporting the active resilience concept^9^,^10^,^11^. Importantly, recent studies have demonstrated that promoting resilience mechanisms achieves treatment efficacy^3^,^7^. These studies open an avenue to a conceptually innovative treatment for depression. However, no druggable targets have been identified so far to potentiate the active resilience mechanisms.

With the goal of therapeutically potentiating this active ionic mechanism, we aimed to identify a behaviourally essential component of the upregulated K^+^ currents in resilient mice to utilize as a possible drug target. In our previous microarray analysis^1^, among the upregulated channels in the VTA of resilient mice that have therapeutic potential as a drug target, we found KCNQ3 (Kv7.3), a slow voltage-activated K^+^ channel^12^,^13^. Consistent with the microarray study indicating KCNQ3 upregulation, we previously functionally demonstrated a remarkable increase in this sustained current in the resilient phenotype^3^. Further, *in vivo* intra-VTA blockade of KCNQ channels by XE-991, a KCNQ blocker, in the mice previously classified as resilient exhibited social avoidance behaviours similar to susceptible animals^14^,^15^. Together, these data indicate that KCNQ may be involved in maintaining the stable resilient phenotype and that the normal firing status of VTA DA neurons is dependent on XE-991 sensitive currents. In addition, it is known that these XE-991-sensitive currents include KCNQ2/3 heterodimers that exhibit an 11-fold larger current compared with KCNQ2 or KCNQ3 homomers^12^,^16^. Therefore, the increased KCNQ3 seen in resilient mice^1^ may play a functional role in stabilizing VTA neuronal activity at the cellular level^17^,^18^.

## Results

### KCNQ3 overexpression normalizes depressive phenotype

**Figure 1:**
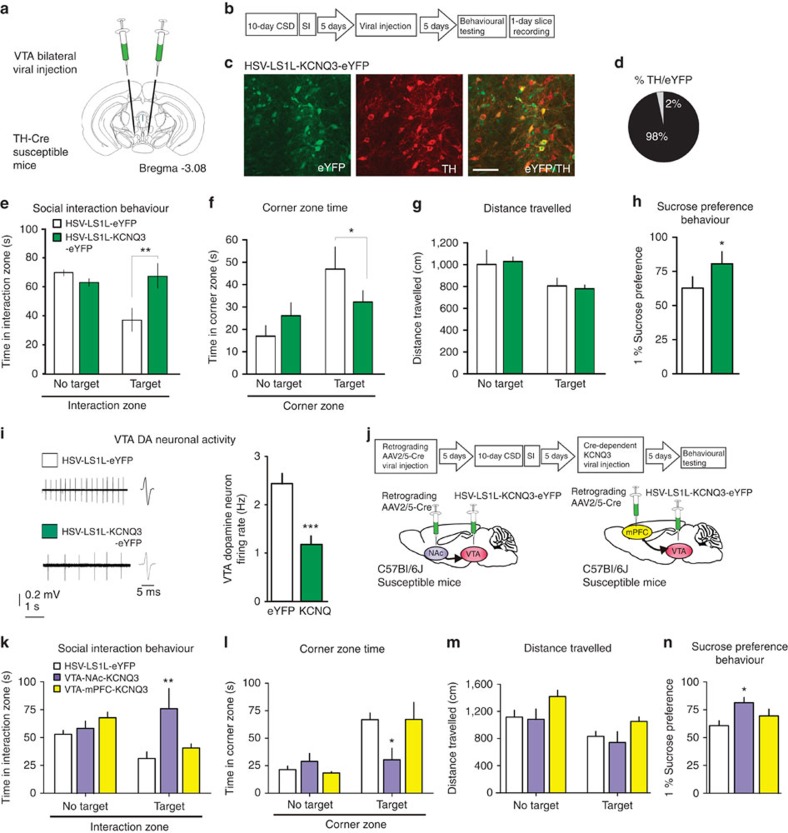
VTA DA neuron- and VTA-NAc-specific overexpression of KCNQ3 channels normalizes depression-like behaviours and neuronal hyperactivity. (**a**,**b**) *In vivo* experimental design. (**c**) Confocal image showing co-expression of HSV-LS1L-KCNQ3-eYFP expression (green) in VTA DA neurons (red) of TH-Cre susceptible mice (scale bar, 50 μm). (**d**) Quantification shows that 98±0.6% of KNCQ3-eYFP-expressing cells are TH^+^ (2–3 sections per mouse from three mice). (**e**) HSV-LS1L-KCNQ3-eYFP viral expression in VTA DA neurons in susceptible mice increases social interaction time with target (*t*_18_=3.88, ^**^P<0.01, *n*=10) and (**f**) decreases corner zone time (*t*_18_=2.63, *P< 0.05, *n*=10) compared with HSV-LS1L-eYFP control virus. (**g**) There is no effect on locomotor activity (*t*_18_=0.80, *P*=0.43, *n*=10). (**h**) Sucrose preference 12 h following social interaction (*t*_18_=3.74, *P<0.05, *n*=10). (**i**) Sample traces and statistic firing data obtained from virally infected VTA DA neurons show a decrease in the HSV-LS1L-KCNQ3-eYFP group compared with the HSV-LS1L-eYFP control virus group (*t*_49_=5.54, ^***^P<0.001, *n*=22–29 cells, 6–8 mice per group). (**j**) Experimental timeline of projection-specific overexpression of KCNQ3 in the VTA and schematic of viral injection of retrograding AAV2/5-Cre into the NAc or mPFC and injection Cre-dependent HSV-LS1L-KCNQ3-eYFP into the VTA of susceptible mice. (**k**) HSV-LS1L-KCNQ3-eYFP viral expression in NAc projecting VTA neurons in susceptible mice increases social interaction time with target (one-way ANOVA: *F*_(2,15)_=4.38, *P<0.05, *n*= 6) and (**l**) decreases corner zone time (*F*_(2,15)_=4.47, *P<0.05, *n*= 6) compared with mPFC projecting VTA neurons or HSV-LS1L-eYFP control virus. (**m**) There is no effect on locomotor activity (*F*_(2,15)_=3.31, *P=0.06, *n*= 6). (**n**) Sucrose preference 12 h following social interaction (*F*_(2,15)_=4.47, *P<0.05, *n*= 6). Mean±s.e.m.

To selectively investigate the KCNQ as a potential therapeutic target through promoting resilience, we used a viral-mediated gene therapy strategy to directly test the hypothesis that an increase in KCNQ-mediated current would relieve depressive symptoms in the susceptible (depressed) mice. To this end, we overexpressed KCNQ3, which is known to form heteromers with KCNQ2 and produce a stabilizing effect on neuronal activity as stated above, selectively in the VTA DA neurons of susceptible mice and tested whether it can induce an antidepressant effect. To restrict the expression of KCNQ3 channels in VTA DA neurons, we used TH-Cre mice and Cre-inducible loxP-STOP-loxP herpes simplex virus (HSV-LS1L-KCNQ3-eYFP), while using HSV-LS1L-eYFP as control. The viral vectors were stereotaxically injected bilaterally into the VTA of TH-Cre susceptible mice (Fig. 1a,b), and morphological validation showed cell type-specific expression (Fig. 1c,d). Our further *in vivo* experiments showed that the previously susceptible mice expressing KCNQ3 spend more time interacting with a social target in the interaction zone (Fig. 1e), and consistently, less time in the corner zone (Fig. 1f), without altering overall locomotor activity (Fig. 1g). Sucrose preference was increased significantly following KCNQ3 expression in the susceptible mice, further supporting the antidepressant effect of KCNQ3 channel overexpression (Fig. 1h). Following behavioural testing, *in vitro* slice recordings revealed that KCNQ3-expressing neurons had significantly lower VTA DA neuron firing activity (Fig. 1i) as compared with the eYFP control group.

Given the functional heterogeneity of VTA projections^2^,^19^,^20^, we next performed viral upregulation of KCNQ3 in the VTA neurons that project either to the nucleus accumbens (NAc) or to the medial prefrontal cortex (mPFC). To achieve this, we utilized a duel viral approach to upregulate KCNQ3 in a projection-specific manner. We injected a retrograding AAV2/5-Cre into either mPFC or the NAc and expressed a Cre-dependent HSV-LS1L-KCNQ3-eYFP in the VTA of susceptible mice (Fig. 1j). This allows for the selective expression in the VTA-NAc or VTA-mPFC pathway (Supplementary Fig. 1). Following expression of KCNQ3, in the VTA-NAc pathway, but not the VTA-mPFC pathway, of susceptible mice resulted in a reversal of depressive behaviours, with previously socially avoidant mice interacting with the social target and spending less time in the corner (Fig. 1k,l), without altering overall locomotor activity (Fig. 1m). Sucrose preference was increased significantly following KCNQ3 expression in the VTA-NAc but no VTA-mPFC neurons of susceptible mice (Fig. 1n). Furthermore, KCNQ3 expression selectively in GABA neurons of the VTA had no antidepressant action in susceptible mice (Supplementary Fig. 2). These observations highlight the diverse responses of VTA DA neuron populations to stress, and suggests that VTA-NAc DA neurons may play a key role in mediating resilience phenotype. The reversal of behavioural and cellular abnormalities in the key circuit of susceptible mice confirms the functional contribution of KCNQ to resilience, and indicates that KCNQ is a valid target for an antidepressant treatment.

### Intra-VTA infusion of KCNQ openers

**Figure 2:**
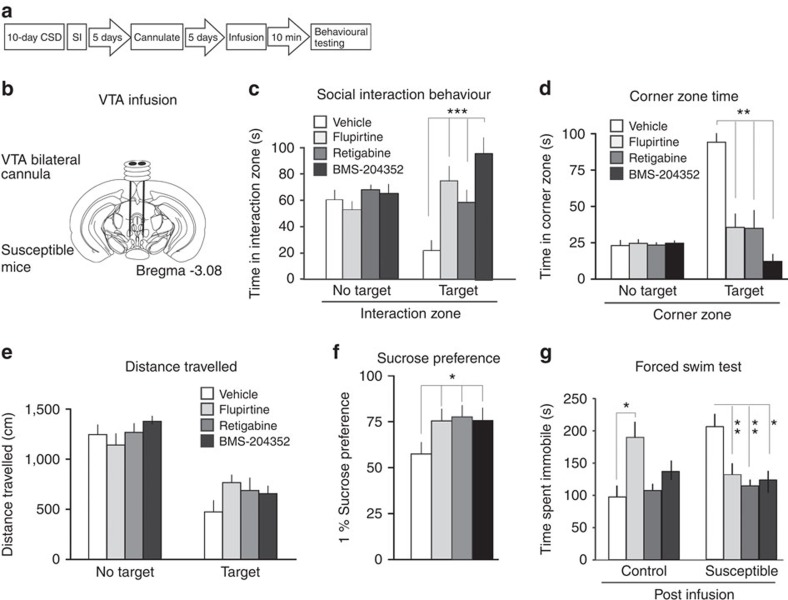
Microinfusion of KCNQ openers flupirtine, retigabine and BMS-204352 into the VTA of susceptible mice consistently normalizes depression-like behaviours. (**a**) Experimental timeline and (**b**) experimental design of VTA bilateral microinfusion into the VTA of susceptible mice. (**c**) Social interaction behaviour is increased significantly in susceptible mice 10 min following acute infusion to the VTA with flupirtine (0.3 μg), retigabine (0.1 μg) or BMS-204352 (0.1 μg), compared with vehicle, indicating an antidepressant-like response (flupirtine: *t*_21_=4.47, ^***^P<0.001; retigabine: *t*_20_=4.71, ^**^P<0.01; BMS-204352: *t*_20_=5.52, ^***^P<0.001; *n*=10–12). (**d**) Corner zone time during social interaction test is significantly reduced compared with vehicle (flupirtine: *t*_21_=3.62, ^**^P<0.01; retigabine: *t*_20_=3.27, ^**^P<0.01; BMS-204352: *t*_20_=5.26, ^**^P<0.01; *n*=9–10). (**e**) Distance travelled during 2.5 min social interaction test (flupirtine: *t*_21_=2.07, *P*=0.11; retigabine: *t*_20_=1.38, *P*=0.18; BMS-204352: *t*_20_=1.28, *P*=0.22; *n*=9–10). (**f**) Sucrose preference measured over a 12-h period after social interaction test (flupirtine: *t*_26_=2.07; retigabine: *t*_26_=2.13; BMS-204352: *t*_26_=2.14; *P<0.05, *n*=14). (**g**) Immobility time is decreased in the forced swim test following infusion to the VTA in susceptible mice indicating an antidepressant-like response following 10 min infusion (flupirtine: *t*_21_=4.15, ^**^P<0.01; retigabine: *t*_21_=3.36, ^**^P<0.01; BMS-204352: *t*_21_=3.61, *P<0.05; *n*=10–13). Mean±s.e.m.

As a potential therapeutic strategy for depression, we investigated currently available pharmacological agents that potentiate KCNQ channels. First, we validated in VTA brain slice preparation the function of three KCNQ channel openers^21^,^22^,^23^, including flupirtine, retigabine and BMS-204352. All three agents when bath applied to *in vitro* slice preparation dose-dependently decreased the hyperactivity observed in the VTA DA neurons of susceptible mice (Supplementary Fig. 3). To directly test if these KCNQ openers could reverse depressive behaviours, susceptible mice received cannulas into the VTA and were behaviourally tested following a single intra-VTA microinfusion of flupirtine (0.3 μg), retigabine (0.1 μg) or BMS-204352 (0.1 μg) (Fig. 2a,b). We found that increasing KCNQ channel function using these pharmacological agents increased time spent with the social target in the interaction zone, reversing the social avoidant behaviour in the previously susceptible mice (Fig. 2c). This was complemented by a corresponding decrease in the amount of time spent in the corner zone (Fig. 2d) with no overall change in locomotor activity (Fig. 2e). A reversal of depression-related behaviours was also observed post infusion as assessed by an increase in sucrose preference (Fig. 2f). Finally, during the forced swim test (FST), a widely used animal test predictive of antidepressant action, the time spent immobile decreased in susceptible mice that were infused with pharmacological KCNQ openers (Fig. 2g). Together, these consistent antidepressant effects strongly support that upregulation of KCNQ channel functions in the VTA is a valid approach to potentiate the active resilience mechanism, and that KCNQ channels are a potential druggable target for depression treatment.

### Systemic administration of KCNQ opener retigabine

**Figure 3:**
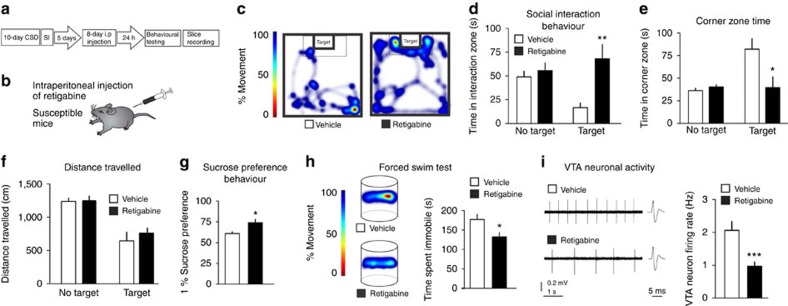
Repeated systemic administration of KCNQ opener retigabine in susceptible mice normalizes depressive behaviours. (**a**) Experimental timeline and (**b**) experimental design of repeated intraperitoneal (i.p.) injections of retigabine in susceptible mice. (**c**,**d**) Social interaction behaviour post repeated i.p. injections of retigabine in susceptible mice shows an increase in the time spent in the interaction zone (*t*_16_=4.02, ^**^P<0.01, *n*=9) and (**e**) a decrease in time spent in the corner zone (*t*_16_=2.65, *P<0.05, *n*=9) compared with vehicle. (**f**) No adverse effects on locomotor activity (*t*_16_=1.23, *P*=0.24, *n*=9). (**g**) Sucrose preference increased following repeated i.p. retigabine when compared with vehicle injections (*t*_10_=2.79, *P<0.05, *n*=6). (**h**) Following repeated i.p. injections of retigabine there is a significant reduction of immobility time during the forced swim test in susceptible mice (*t*_10_=2.48, *P<0.05, *n*=6). (**i**) The hyperactivity of VTA neurons is normalized in susceptible mice (*t*_66_=3.64, ^***^P<0.001, *n*=34 cells, 7 mice per group). Means±s.e.m.

To further investigate the therapeutic potential of KCNQ channels, we systemically administered the KCNQ opener retigabine in susceptible mice. Among the available KCNQ pharmacological openers, retigabine (ezogabine), which stabilizes the open state of the channel^24^,^25^, is an FDA-approved agent. Its primary indication is as an anticonvulsant, marketed under the name Potiga. Given its current availability and efficacy as a KCNQ channel opener in models of epilepsy, chronic pain and mania^26^,^27^, we systemically administered retigabine to socially defeated susceptible mice and performed behavioural testing (Fig. 3a,b). Following eight repeated but not single (Supplementary Fig. 4) intraperitoneal injection (i.p.) of retigabine, susceptible mice spent more time interacting with the social target in the interaction zone (Fig. 3c,d) and less time in the corner zone (Fig. 3e), without adverse effects on locomotor activity (Fig. 3f). Chronic systemic administration of retigabine increased sucrose preference and decreased immobility time during the FST in susceptible mice when compared with vehicle treatment, further indicating the antidepressant effects of retigabine (Fig. 3g,h). In contrast, repeated i.p. injections of retigabine in stress-naive mice had no adverse behavioural effects (Supplementary Fig. 5). Following behavioural testing of susceptible mice, *in vitro* VTA slice recordings were performed to determine if the cellular functions were regulated as a result of retigabine administration. VTA DA neurons of susceptible mice that received repeated systemic administration of retigabine demonstrated a normalization of firing rate as compared with the vehicle-injected mice (Fig. 3i). These results provide a direct systemic validation of retigabine as a potential antidepressant that potentiates the active ion channel mechanism of natural resilience.

## Discussion

Overall, this study provides a therapeutic strategy of using active resilient mechanisms to normalize the pathogenic hyperactivity of VTA DA neurons. It is known that DA neurons in the brain's reward system have a constant baseline firing to provide a necessary level of dopamine to their target brain regions. This baseline activity is altered differentially or even oppositely in distinct stress models of depression such as severe social defeat stress and mild non-social stress paradigms^1^,^3^,^8^,^28^,^29^,^30^,^31^,^32^,^33^, and these opposite pathological alterations in VTA DA neurons have been causally linked to depression-related behaviours in recent optogenetic studies^2^,^32^. These opposing effects of different stress models are not unexpected given the complex and diverse aetiology of human depressive symptoms, with frequent extremes in symptomology being expressed. These extremes are highlighted in human depression studies in which, insomnia/hypersomnia, anorexia/overeating are among the conflicting symptomology^34^. In the well-established chronic social defeat model, the pathogenic hyperactivity of VTA DA neurons is, in part, driven by intrinsic changes in channel function. These changes maybe unique to aetiology of symptoms expressed. Specifically, an increased *I*_h_ current (hyperpolarization-activated cation channel-mediated current) is seen in susceptible mice^3^,^8^, whereas the stable firing found in the resilient group is homeostatically maintained by counteracting this pathogenic *I*_h_ current with K^+^ channels^3^. Our current data support the KCNQ contribution to these active K^+^ channels in resilience. Importantly, directly potentiating KCNQ channels in susceptible mice normalizes the pathogenic hyperactivity of VTA DA neurons, and has significant antidepressant effects as assessed at the behavioural level. Our study identifies KCNQ channels as a novel target to stabilize VTA DA neurons and achieve treatment efficacy following severe conditions of social stress. It is intriguing for the future work to explore whether enhancing active resilience mechanisms such as retigabine has a protective effect before the onset of depression or during the induction of depression.

From a clinical-translational perspective, retigabine is currently FDA approved for use as anticonvulsant for partial epilepsies, indicating that it may exert its treatment effects by targeting pathological regions of hyperactivity in the brain. Retigabine's powerful normalization of the pathogenic hyperactivity of VTA DA neuron activity is a locus for an anti-depressive therapeutic effect, although it may not be the only site of efficacy. In addition to retigabine's use for treatment of partial-onset seizures, recent research has indicated it has a wide array of potential uses ranging from treating amyotrophic lateral sclerosis, chronic pain to alcohol addiction^35^,^36^,^37^,^38^. Although potential negative effects of systemic KCNQ potentiation in patients have to be considered, our studies identify KCNQ channels as a promising previously uncharacterized target in treatment of major depressive disorder. Overall, our data support the concept of an ‘active' antidepressant that utilizes active ionic resilience mechanisms and thereby pave the way for a novel, urgently needed treatment strategy for patients with depression.

## Methods

### Mice

This study used TH-BAC-Cre^39^, and GAD2-IRES-Cre^40^ mice with C57BL/6J gene background that were bred at Icahn School of Medicine at Mount Sinai and used at 8 weeks, the age at start of experimental manipulations. Wild-type 7-week-old male C57BL/6J mice were ordered from Jackson Laboratory for some studies. These mice were acclimated to the housing facility for 1 week prior to experiments. As in our previous studies^1^,^2^,^3^, all mice were group housed and maintained on a 12-h light, 12-h dark cycle with *ad libitum* access to food and water until start of experiments. Following chronic social defeat stress, mice were then singly housed and maintained on a 12-h light, 12-h dark cycle with *ad libitum* access to food and water. All experiments performed are approved by the Institutional Animal Care and Use Committee and comply with institutional guidelines for Animal Care and Use Committee set forth by Icahn School of Medicine at Mount Sinai.

### Chronic social defeat stress paradigm

Chronic social defeat stress was performed according to published protocols^1^,^2^,^3^,^41^. Briefly, CD1 aggressive mice were housed in social defeat cages (26.7 cm width × 48.3 cm depth × 15.2 cm height; Allentown Inc.) 24 h before the start of defeats on one side of a clear perforated Plexiglass divider. During each defeat episode, C57BL/6J or TH-BAC-Cre mice were allowed to interact for 10 min with an aggressive CD1 mouse, during which these mice undergo a physical bout of interaction. For the remainder of the day, the C57BL/6J or TH-BAC-Cre mouse is then housed across a clear plexiglass divider providing further sensory cues. After 10 bouts of defeat occurring, over 10 consecutive days the C57BL/6J or TH-BAC-Cre mice were singly housed. Mice underwent the social interaction test 24 h after the final defeat episode. Control mice were housed two mice per cage divided by a perforated plexiglass divider and rotated similar to the defeat mice.

### Social interaction test

Social interaction testing was performed as previously described^1^,^2^,^3^,^4^,^41^. The social interaction test, measured the time spent in the interaction zone, corner zones and locomotor activity during the first (CD1 target absent in interaction zone) and second (CD1 target present in interaction zone) trials in an open-field arena. Their movements were automatically monitored and recorded (Ethovision 10.0, Noldus Information Technology) for 2.5 min each test session. Behavioural phenotyping was performed on day 11. The segregation of susceptible and resilient mice was based on the social interaction ratio, which was calculated as (100 × (interaction time, target present)/(interaction time, target absent)) as described previously^1^,^2^,^3^,^41^. Social interaction behaviour was then calculated as total time spent in each zone or as a ratio of the time spent in the interaction with target present divided by the time spent in the interaction zone with the target absent. All mice with a ratio above 100 were classified as resilient, and all mice below 100 were classified as susceptible.

### Sucrose preference

To determine if mice developed anhedonic responses to the experimental manipulations, a sucrose preference test was performed^1^,^2^,^3^. For sucrose-preference testing, a solution of 1% sucrose or drinking water was filled in 50 ml tubes with stoppers fitted with ball-point sipper tubes (Ancare). All animals were singly housed and acclimatized to two-bottle choice conditions prior to testing conditions. Twelve hours post social interaction, fluid was weighed, and the positions of the tubes were interchanged. Sucrose preference was calculated as a percentage (100 × volume of sucrose consumed (in bottle A)/total volume consumed (bottles A and B)). The preference for sucrose over water is a measure of anhedonic reward sensitivity.

### Forced swim test

The FST was performed as previously described^3^. Ten minutes following infusion mice were placed for 6 min in a 4-l Pyrex glass beaker containing 3 l of water at 24±1 °C. The water was changed between test subjects. All test sessions were recorded by a digitally tracking Ethovision program, positioned on the side of the beaker to record mouse movements. Time spent immobile was independently analysed by Ethovision 10.0 software. Increased immobility time and decreased latency to immobility is a measure of behavioural despair. Sample heatmap of activity was automatically generated in Ethovision 10.0 (Noldus Information Technology).

### Electrophysiology

Acute coronal brain slices of VTA were prepared according to previously published protocols^2^,^3^. All recordings were carried out blind to drug treatment. Male 8–12-week-old mice were perfused with cold artificial cerebrospinal fluid (aCSF) containing (in mM): 128 NaCl, 3 KCl, 1.25 NaH_2_PO_4_, 10 D-glucose, 24 NaHCO_3_, 2 CaCl_2_ and 2 MgCl_2_ (oxygenated with 95% O_2_ and 5% CO_2_, pH 7.35, 295–305 mOsm). Acute brain slices containing the VTA were cut using a microslicer (DTK-1000, Ted Pella) in sucrose-ACSF, which was derived by fully replacing NaCl with 254 mM sucrose, and saturated by 95% O_2_ and 5% CO_2_. Slices were maintained in the holding chamber for 1 h at 37 °C. Slices were transferred into a recording chamber fitted with a constant flow rate of aCSF equilibrated with 95% O_2_ and 5% CO_2_ (2.5 ml min^−1^) and at 35 °C. Glass recording pipettes (2–4 MΩ) were filled with an internal solution containing (mM): 115 potassium gluconate, 20 KCl, 1.5 MgCl_2_, 10 phosphocreatine, 10 HEPES, 2 magnesium ATP and 0.5 GTP (pH 7.2, 285 mOsm). VTA DA neurons were identified by their location and infrared differential interference contrast microscopy and recordings were made from TH positive neurons in eYFP virally tagged neurons. Firing rate was recorded in the cell-attach mode, and data acquisition and on-line analysis of firing rate were collected using a Digidata 1440A digitizer and pClamp 10.2 (Axon Instruments). The sequence and timing of recordings is consistent throughout treatment groups.

### Immunohistochemistry

Mice were perfused with 30 ml cold PBS and 30 ml 4% paraformaldehyde (PFA). The brain were taken out and post-fixed with same fixative overnight, then treated with 30% sucrose at 4 °C for 2 days. Brain tissue was sectioned with thickness of 30 μm and stored in at 4 °C till use. Brain sections were rinsed with PBS and blocked with blocking buffer (BSA, PBS with 0.2% Triton X-100) for 1 h. Sections were incubated with primary Anti-TH monoclonal antibody (Sigma 1:10,000) and anti-GFP (Invitrogen 1:1,000) at 4 °C. Next day, sections were incubated with secondary (Alexa Fluor 488 and Cy5) for 1 h. Then rinse with PBS three times before mounting. Z-stack, tile scans and single images were acquired using Zeiss 880 laser scanning confocal microscope equipped with a 32-spectral array GaAsp detector (Carl Zeiss, Jena, Germany) using × 20 Plan-Apochromat objective. eYFP labelled with Alexa 488 antibody was excited using 488 nm argon ion laser line and the fluorescence emission was collected from 491 to 600 nm. Alexa 647 was excited using a 633-nm HeNe laser line and the fluorescence emission was collected from 638 to 759 nm. Images presented are maximum projection intensity images from 20 z-slices with a thickness 1.0 μm per slice. Images were analysed using the ZEN 2014 software.

### Cannula implantation

Cannula implantation surgeries were performed as described in our previous work^3^,^6^. Mice were anaesthetized by the intraperitoneal injection of a mixture ketamine (100 mg kg^−1^) and xylazine (10 mg kg^−1^) anaesthesia and placed into a stereotaxic apparatus (David Kopf Instruments, Tujunga, California). Mice were bilaterally implanted with a stainless 26 gauge cannulae fitted with obturators (Plastics One) targeting directly above the VTA with the following coordinates: anteroposterior −3.3 mm; lateral 0.5 mm; dorsoventral −3.7 mm. The cannulae were secured with dental cement (Dentsply); the mice were then singly housed and allowed to recover for 5 days.

### VTA microinfusion

Following cannuale surgery and recovery mice received an acute intra-VTA infusion of XE-991, flupirtine, retigabine, BMS-204352 or filtered 1 × PBS as vehicle control. All drugs were prepared daily and are available for commercial purchase (Tocris, Alomone, Sigma). Simultaneous bilateral microinjections were delivered through an injector cannula in a total volume of 0.4 μl per side at a continuous rate of 0.1 μl min^−1^ under the control of a microinfusion pump (Harvard Apparatus). Concentrations were selected based on earlier *in vivo* studies^21^,^26^. Injectors were removed 5 min after the stopping of each infusion, and mice remained undisturbed in their home cages prior to social interaction behaviour. All cannulae placements were confirmed post mortem.

### Stereotaxic surgery and viral gene transfer

We performed all surgeries under aseptic conditions using anaesthetic as described previously^2^,^3^,^6^. TH-Cre mice were anaesthetized with a mixture of ketamine (100 mg kg^−1^) and xylazine (10 mg kg^−1^), positioned in a small-animal stereotaxic instrument (David Kopf Instruments), and the skull surface was exposed. To selectively target DA neurons in the VTA, we utilized a combination of TH-Cre mice and a Cre inducible HSV-LSIL-KCNQ3-eYFP virus to overexpress the KCNQ3 channels, similar to our previous studies. To selectively target neurons in the VTA that project to the Nucleus accumbens or medial prefrontal cortex, we utilized a duel virus approach in C57BL/6J mice. A combination of a retrograding AAV2/5-Cre virus injected into the NAc (bregma coordinates: AP, +1.5; LM, +1.6; DV, –4.4; 10° angle) or mPFC (bregma coordinates: AP, +1.7; LM, +1.6; DV, –2.5; 15° angle) and a Cre inducible HSV-LSIL-KCNQ3-eYFP virus to overexpress the KCNQ3 channels in the VTA. Therefore, KCNQ3 was expressed selectively in NAc or mPFC projecting neurons in the VTA. These viruses were provided by Rachael Neve's laboratory in Massachusetts Institute of Technology (MIT) and the University of Pennsylvania vector core. Thirty-three-gauge syringe needles (Hamilton Co.) were used to bilaterally infuse 0.5 μl of virus HSV-LS1L-KCNQ3-eYFP or HSV-LS1L-eYFP into the VTA at a 7° angle (bregma coordinates: AP, +3.3 mm; LM, +1.0 mm; DV, −4.6 mm) at a rate of 0.1 μl min^−1^. All behavioural analyses were conducted days 3–4 after infusion during maximal HSV expression.

### Retigabine treatment

After social interaction testing, mice were sorted into either susceptible or resilient phenotypes on the basis of their social interaction ratio as described above. Control mice were housed identically to defeated mice for the duration of the experiment but were never exposed to social defeat sessions. Susceptible and control subgroup mice were used and randomly divided into treatment groups. For each group (control or susceptible), mice received either daily i.p. injections of retigabine (1 mg kg^−1^ body weight) for 8 days or vehicle i.p. injections. All mice then underwent a social interaction test 24 h post final injection. Immediately following the social interaction test, one subset of mice underwent sucrose preference, one subset underwent FST and a third group underwent VTA slice recordings with measurements of firing rate.

### Statistics

All analyses were performed with Prism software. Comparisons were achieved by means of analyses of variance with/without repeated factors (ANOVA; multiple-groups comparisons). In the latter case, *post hoc* comparisons (Tukey) were performed when appropriate. When appropriate, a two-way Student's *t*-tests were used to determine statistical significance of planned comparisons. Data are expressed as the mean±s.e.m. Statistical significance was set at *P*<0.05.

### Data availability

All relevant data are included within the manuscript's figures or in supplementary files. Any additional data is available upon request from the corresponding author.

## Additional information

**How to cite this article:** Friedman, A. K. *et al*. KCNQ channel openers reverse depressive symptoms via an active resilience mechanism. *Nat. Commun.* 7:11671 doi: 10.1038/ncomms11671 (2016).

## Supplementary Material

Supplementary Information Supplementary Figures 1-5
