# Supplementary material for: KCNQ channel openers reverse depressive symptoms via an active resilience mechanism
Source: Nat Commun. 2016 May 24;7:11671. doi: 10.1038/ncomms11671 (PMC4890180; doi:10.1038/ncomms11671)
Supplement: Supplementary Information — Supplementary Figures 1-5 [file ncomms11671-s1.pdf]

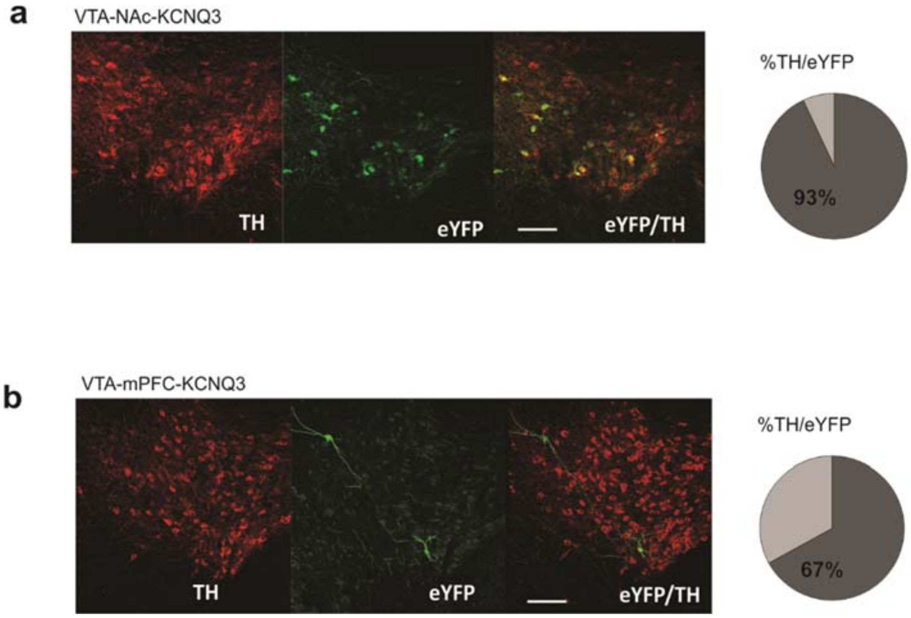

Supplementary Figure 1 | Nucleus accubens (NAc) and medial prefrontal cortex (mPFC) projecting neurons of the ventral tegmental area (VTA) expressing HSV-LS1L-KCNQ3-eYFP. (**a**) Confocal images of co-expression of HSV-LS1L-KCNQ3-eYFP expression (green) in NAc projecting VTA TH^+ neurons (red) and quantification demonstrating a 93% ± 0.3% of KCNQ3 expressing neurons are TH^+ (scale bar 50 μm). (**b**) Confocal images of co-expression of HSV-LS1L-KCNQ3-eYFP expression (green) in mPFC projecting VTA TH^+ neurons (red) and quantification demonstrating a 67% ± 0.5% of KCNQ3 expressing neurons are TH^+ (3-4 sections per mouse from 3 mice per projection) (scale bar 50 μm).

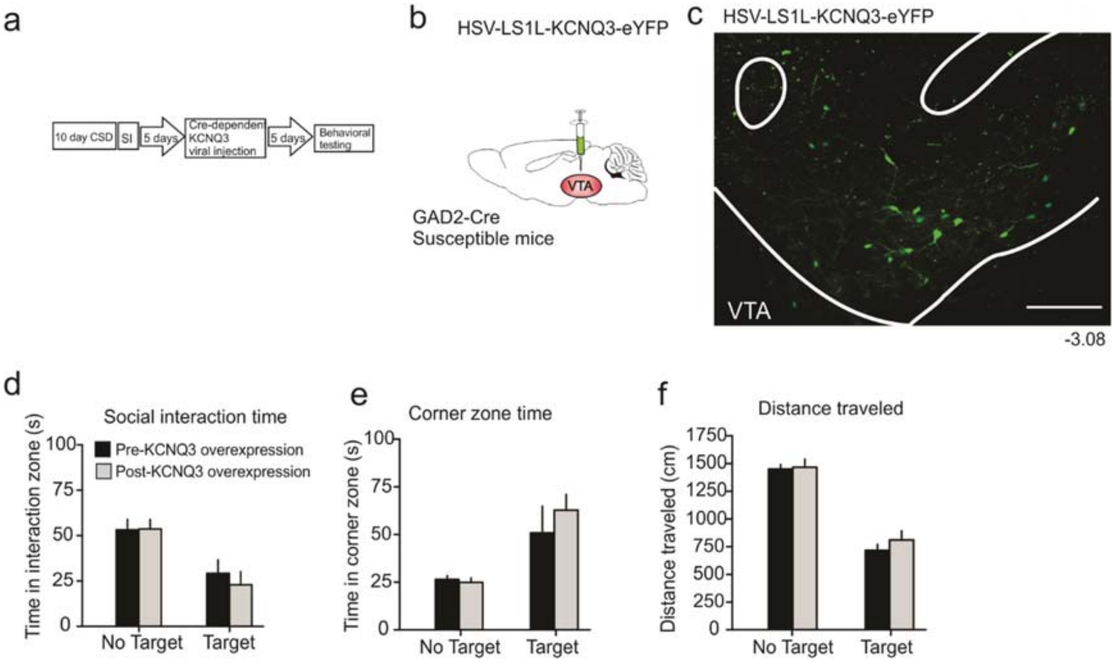

Supplementary Figure 2 | Overexpression of KCNQ3 channels selectively in the VTA GABA neurons of susceptible GAD2-IRES-Cre mice does not alter depressive like-behaviors. (**a**) Experimental timeline. (**b,c**) Schematic and confocal image of viral injection of HSV-LS1L-KCNQ3-eYFP expression (green) into the VTA of susceptible GAD2-IRES-Cre mice (scale bar 50 μm). (**d**) Social interaction behavior post HSV-LS1L-KCNQ3-eYFP expression in susceptible mice shows no change in the time spent in the interaction zone (paired t-test: t_7 =0 .52, p = 0.62, n = 8) and (**e**) no change in time spent in the corner zone (t_7 = 0.65, p = 0.54, n = 8) as compared to pre-expression. (**f**) There are no effects on locomotion with target present (t_7 = 0.92, p = 0.39, n = 8).

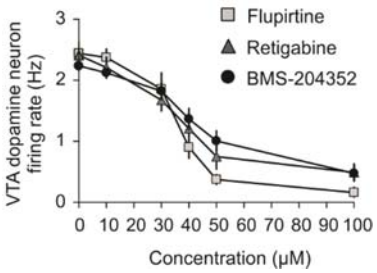

Supplementary Figure 3 | Flupirtine, retigabine and BMS-20435 when bath applied to *in vitro* slice preparation dose-dependently decreased the hyperactivity observed in the VTA DA neurons of susceptible mice as measured by cell-attached firing rate recordings.

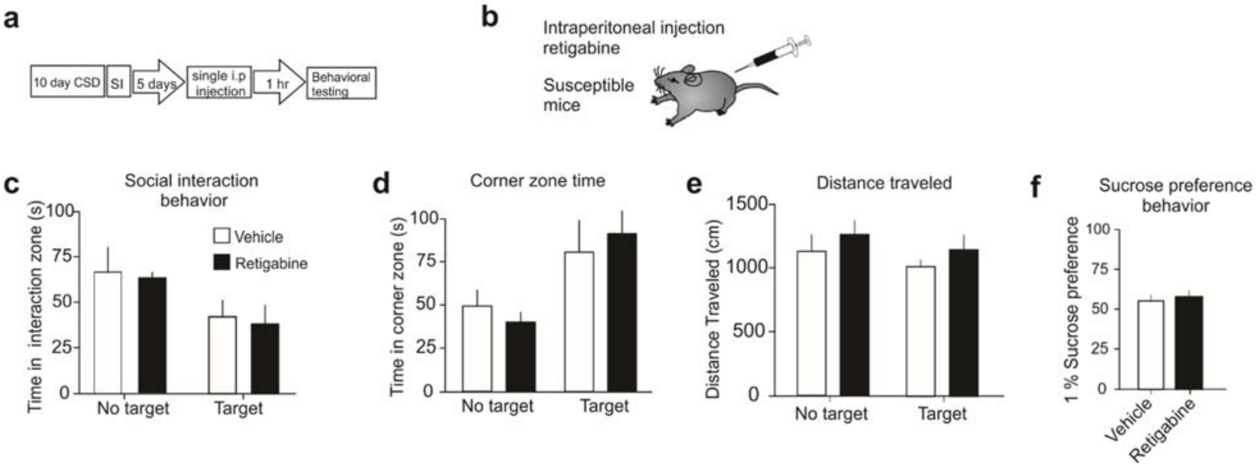

Supplementary Figure 4 | Single systemic administration of KCNQ channel opener retigabine does not alter social avoidance behavior in susceptible mice. (**a**) Experimental timeline and (**b**) experimental design of single i.p. injection of retigabine in susceptible mice. (**c**) Social interaction behavior post repeated i.p. injection of retigabine in susceptible mice shows no change in the time spent in the interaction zone (t_8 =0 .53, p = 0.53, n = 5) and (**d**) no change in time spent in the corner zone (t_8 = 0.46, p = 0.65, n = 5) as compared to vehicle group. (**e**) There are no effects of one dose of retigabine injection on locomotion with target present (t_8 = 1.03, p = 0.33, n = 5) or (**f**) sucrose preference when compared to vehicle group in susceptible mice (t_8 = 0.50, p = 0.63, n = 5). Mean ± s.e.m.

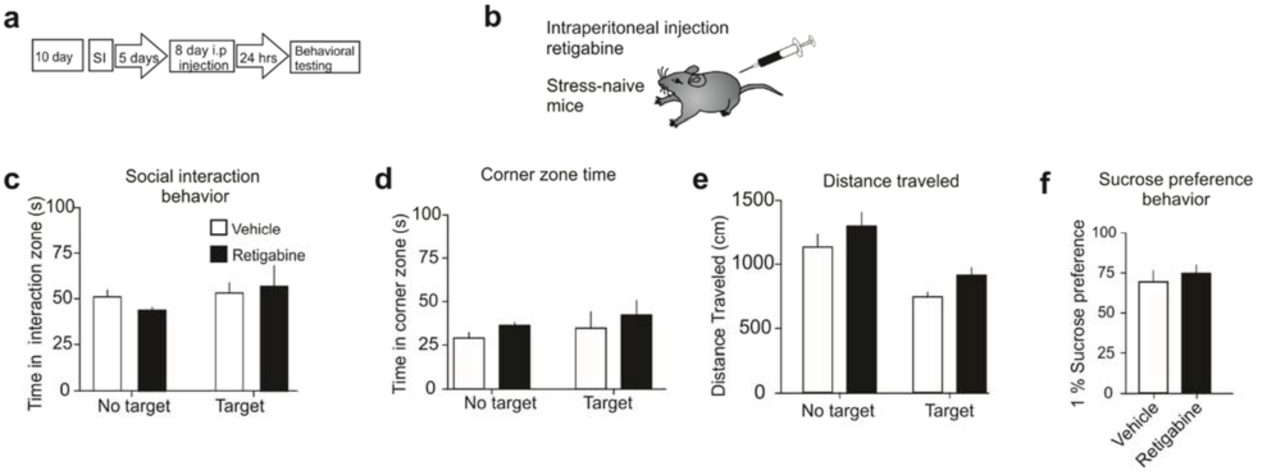

Supplementary Figure 5 | Repeated systemic administration of KCNQ channel opener retigabine has no adverse effects on social interaction behavior in stress-naïve mice. (**a**) Experimental timeline and (**b**) experimental design of repeated i.p. injections of retigabine in stress-naïve mice. (**c**) Social interaction behavior post repeated i.p. injection of retigabine in stress-naïve mice shows no change in the time spent in the interaction zone ($t_8 = 0.35$, $p = 0.73$, $n = 5$) and (**d**) no change in time spent in the corner zone ($t_8 = 0.56$, $p = 0.56$, $n = 5$) as compared to vehicle group. (**e**) There are no adverse effects repeated retigabine injection on locomotion with target present ($t_8 = 1.30$, $p = 0.23$, $n = 5$) or (**f**) sucrose preference compared to vehicle group in stress-naïve mice ($t_8 = 0.90$, $p = 0.39$, $n = 5$). Mean ± s.e.m.
